# Identifying factors which enhance capacity to engage in clinical education among podiatry practitioners: an action research project

**DOI:** 10.1186/s13047-015-0123-4

**Published:** 2015-11-26

**Authors:** Sally Abey, Susan Lea, Lynne Callaghan, Steve Shaw, Debbie Cotton

**Affiliations:** Faculty of Health and Human Sciences, Plymouth University, Plymouth, UK; Deputy Vice-Chancellor (Academic), University of Greenwich, London, UK; Centre for Mental Health and Justice, Cornwall Partnership NHS Foundation Trust, Cornwall, UK; School of Computing and Mathematics, Plymouth University, Plymouth, UK; Pedagogic Research Institute and Observatory, Plymouth University, Plymouth, UK

**Keywords:** Capacity building, Professional education, Podiatry, Psychometrics, Questionnaires

## Abstract

**Background:**

Health profession students develop practical skills whilst integrating theory with practice in a real world environment as an important component of their training. Research in the area of practice placements has identified challenges and barriers to the delivery of effective placement learning. However, there has been little research in podiatry and the question of which factors impact upon clinical educators’ capacity to engage with the role remains an under-researched area. This paper presents the second phase of an action research project designed to determine the factors that impact upon clinical educators’ capacity to engage with the mentorship role.

**Methods:**

An online survey was developed and podiatry clinical educators recruited through National Health Service (NHS) Trusts. The survey included socio-demographic items, and questions relating to the factors identified as possible variables influencing clinical educator capacity; the latter was assessed using the ‘Clinical Educator Capacity to Engage’ scale (CECE). Descriptive statistics were used to explore demographic data whilst the relationship between the CECE and socio-demographic factors were examined using inferential statistics in relation to academic profile, career profile and organisation of the placement.

**Results:**

The survey response rate was 42 % (*n* = 66). Multiple linear regression identified four independent variables which explain a significant proportion of the variability of the dependent variable, ‘capacity to engage with clinical education’, with an adjusted R_2_ of 0.428. The four variables were: protected mentorship time, clinical educator relationship with university, sign-off responsibility, and volunteer status.

**Conclusion:**

The identification of factors that impact upon clinical educators’ capacity to engage in mentoring of students has relevance for strategic planning and policy-making with the emphasis upon capacity-building at an individual level, so that the key attitudes and characteristics that are linked with good clinical supervision are preserved.

## Background

Placement and work-based learning is of increasing importance in higher education as students build practical skills alongside their academic learning [1]. In the United Kingdom (UK), effective clinical education of healthcare professionals is facilitated through formal collaborations between the National Health Service (NHS) and higher education institutions, and students can spend up to half of their programme of study on placement. A key factor in placement success is the role of the clinical educator. The clinical educator can make a difference between a supportive placement in which learning is maximised and one in which the student becomes disengaged and potentially fails to achieve learning outcomes [2–4]. While the term ‘clinical educator’ is generally ill-defined in the literature, in this study the role is seen as similar to that of the ‘nursing mentor’ (UK Nursing and Midwifery Council) who supports learning and assessment in practice across a range of domains [5]. In recent years, the responsibilities of the clinical educator have increased considerably [2, 6] while student numbers have also increased [7] and the focus on quality has sharpened [8, 9].

A range of factors has been shown to impact the student placement experience. These include the level of student preparation for placement; appropriate induction to the placement environment [10]; student numbers [11]; clinical educator’s self-efficacy [10]; and the complexities of the clinical environment, including the clinical educator-student relationship [12]. Despite the importance of the clinical educator relatively little research has examined the factors associated with clinical educators’ engagement with the role. In nursing, studies have shown that mentorship, enthusiasm for the nursing profession and collegiality are important [13]. Research has also identified universities provision of support to the placement area [14, 15], clinical educators obtaining a positive view of students [6] and protected time for mentoring [13] as influential factors.

Recently, research in the UK and Australia has started to focus on how increased numbers of students can be facilitated to undertake practice placement opportunities (a focus on organisational ‘capacity’ [8, 16–22]), driven by changing workforce requirements [23–25], including the need to train more healthcare professionals, particularly nurses, as clinical educators [7, 11, 26]. There are many restrictions on capacity [23, 24], with clear tensions between increasing capacity and the provision of high quality placements [27] and patient care [11].

Research on capacity to date has focused largely on nursing and midwifery, and limited to staff perceptions of clinical education, specifically the negotiation of student numbers. The authors argue for the need to redefine and broaden the concept of capacity in healthcare clinical education. This definition includes building the capacity of significant individuals, groups and organisations to provide sustainable clinical education within the placement environment [28]. A multi-factorial concept, capacity-building requires a whole system approach to understand and support the complex structures which underpin the increase in students allocated to the placement setting. The clinical educator plays a critical part in facilitating students within the clinical environment and their capacity to undertake the role and manage the learning environment requires support and development. Building capacity in this context involves an on-going process which empowers the organisation, and the groups and individuals within it [28] to achieve the objective of effective, high quality placement learning. It is therefore essential to examine the role of the clinical educator within this whole system approach.

The ‘Clinical Educator Capacity to Engage’ scale (CECE) was developed by the authors [17] to identify the variables that predict podiatry clinical educator capacity to engage with the role of mentorship. The 74-item CECE scale comprises nine sub-scales (anxiety; confidence; culture; job satisfaction; leadership; management; support; positive attitude towards the role of clinical educator; negative attitude towards the role of clinical educator) and has been shown to have good reliability [17]. Establishing the factors that impact upon clinical educator capacity may identify opportunities for placement planning, organisation and support, resulting in more effective practice placement. The aim of the research was to survey podiatry clinical educators to explore factors thought to predict the variability of clinical educator capacity to engage in the mentorship role.

## Method

**Fig. 1:**
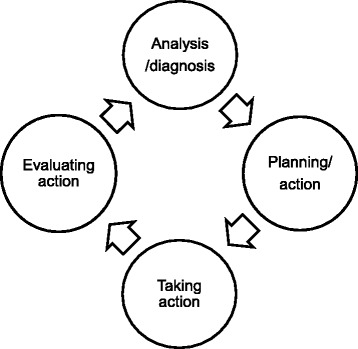
Spiral of action research cycles adapted from Coghlan and Brannick [29]

This research represents part of a larger collaborative action research project between one higher education institute and an NHS podiatry department. An action research approach allowed for the exploration of the complex issues that surround placement learning whilst taking a collaborative approach with stakeholders [29]. The framework supports a systematic approach to problem-solving [30] where issues/challenges/barriers are ‘analysed/diagnosed’, which leads to the formulation of an ‘action plan’ which addresses issues and changes practice (see Fig. 1). The action can subsequently be evaluated by the whole action research team and is a powerful way of informing practice where mixed methodological approaches to research may be applied [31].

### Action research team

The stakeholders forming the action research team comprised clinical educators from podiatry and nursing. These stakeholders discussed the barriers and challenges to clinical education and provided a multi-lens perspective of this complex environment. The initial ‘diagnosis phase’ (cycle one) had established that the team held positive attitudes towards the clinical educator role and regarded it as an integral part of their professional responsibility as a healthcare professional. From these discussions, the construct ‘*capacity to engage*’ was generated and broadly defined. The Action Research Team (ART) felt the clinical educators’ capacity to engage with clinical education was high, but a lack of empirical research meant that this belief could not be substantiated.

### Capacity to engage with clinical education scale

During the planning phase the ART and SA, SL and LC developed an instrument for measuring Clinical Educators’ Capacity to Engage with the mentorship role, the *CECE* scale [23], consisting of 74 items within nine sub-scales: anxiety; confidence; culture; job satisfaction; leadership; management; support; positive attitude towards the role of clinical educator; negative attitude towards the role of clinical educator.

The scale has subsequently been found to have good to excellent reliability (Cronbach’s alpha 0.782 to 0.951) following the piloting with podiatry clinical educators within 25 English NHS Trusts. The development and piloting of the scale was an important first step to ascertaining reliability.

### Workshop 1: Research model and independent variables

**Fig. 2:**
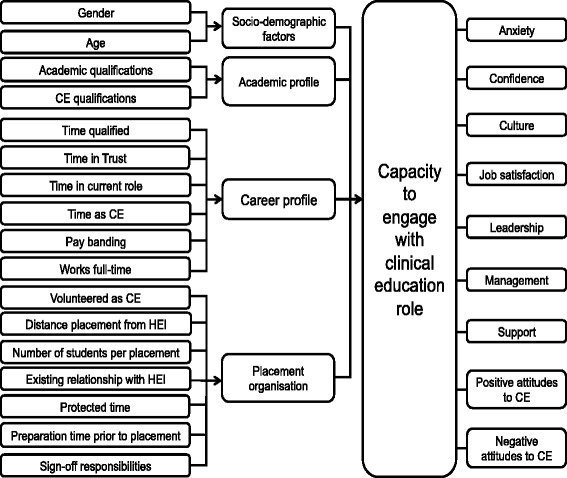
Independent variables shown in relation to dependent variable: Capacity to engage with the role of clinical education

The ART initially met with SA to discuss the development of the scale alongside identifying the potential factors that might impact upon an individual’s capacity to engage with clinical education. As part of the discussion, factors (variables) that were thought to impact upon this capacity were identified from the considerable combined pedagogical experience of the clinical educators within the team, alongside nursing and midwifery literature. These predictive factors were reworked into hypotheses that could then be tested against the CECE scale (see Fig. 2). The independent variables that were identified as potentially influential were socio-demographic factors, academic profile, career profile, and placement organisation.

### Ethics

Ethical approval was granted by the Cornwall & Plymouth Research Ethics Committee (09/H0203/95) as well as the Plymouth University Ethics Committee.

## Data collection

### Recruitment

Heads of service in 15 podiatry departments were sent a letter inviting staff who act as clinical educators to participate in the research. Postcards were included which advertised the research and offered potential participants the opportunity to win one of two £25 book vouchers on completion of the survey. Participant anonymity was assured, although an email address was requested if individuals wished to be entered into the prize draw; this was administered by an independent third party.

### Materials

The survey was hosted online and heads of service were asked to engage their staff in the research by forwarding the postcard to them. The survey was live for a six-week period and at two weekly intervals reminder emails were issued.

### The Sample

The population for the research comprised all podiatrists with clinical educator responsibilities, regularly or on an ad hoc basis, for the BSc (Hons) Podiatry programme at one UK University.

### Data analysis

All analysis was undertaken using PASW® version 18. Descriptive statistics were used to examine demographic data. The relationship between the CECE scale and socio-demographic factors, academic profile, career profile, and placement organisation was examined. Analysis was restricted to the use of non-parametric tests where the data were ordinal and nominal in nature. Inferential statistics were used, specifically the Mann–Whitney U and Kruskal-Wallis tests leading to multiple linear regression. Individual factors were explored and those that were significant informed a regression which sought to identify a model of clinical educator capacity. A significance level of 0.05 was set (See Table 1) with an adjustment of the p-value threshold to <0.1 as stated in Table 2. Assumptions of linearity and homoscedasticity were met overall in relation to the multiple linear regression [30].

**Table 1:** Factors associated with podiatrists’ capacity to engage in clinical education

Factor	Statistical test	N	Results	Significance
Participants who volunteer as clinical educators demonstrate greater capacity to engage	Mann–Whitney U	66	U = 306.0	*p* = 0.003
A relationship with the university, outside the clinical educator role (e.g. previous student) will produce greater capacity to engage in the role	Mann–Whitney U	65	U = 253.5	*p* = 0.099*
Clinical educators’ engagement with the role increases when employed full-time	Mann–Whitney U	60	U = 260.5	*p* = 0.010
Preparation time prior to student attendance on placement would increase capacity to engage	Mann–Whitney U	59	U = 132.5	*p* = 0.002
Where protected time outside clinical hours was timetabled the hypothesis stated that capacity to engage would be higher	Mann–Whitney U	59	U = 115.0	*p* = 0.002
Clinical educators with only a single student to mentor per placement would show greater capacity for engagement	Mann–Whitney U	66	U = 361.5	*p* = 0.037
Responsibility for signing-off students’ learning outcomes would impact positively on clinical educators’ capacity to engage	Mann–Whitney U	65	U = 248.0	*p* = 0.006
Where clinical educators’ employment was closer to the university capacity scores would be higher. Five distance categories were established: 0 to 49 miles; 50 to 99 miles; 100 to 149 miles; 150 to 199 miles; 200 to 249 miles	Kruskal-Wallis	65	H(4) = 8.78	*p* = 0.067*

*significance level was raised to *p* ≤ 0.1

**Table 2:** Factors not associated with podiatrists’ capacity to engage in clinical education

Factors	Statistical test	N	Results	Significance
The length of time a clinical educator has been qualified as a podiatrist will results in higher capacity to engage scores	Spearman’s rho	65	r_s_ = 0.119	*p* = 0.346
The length of time a clinical educator has worked for a particular NHS Trust will result in higher capacity to engage scores	Spearman’s rho	66	r_s_ = 0.173	*p* = 0.165
The length of time a clinical educator has worked in a particular role will result in higher capacity to engage scores	Spearman’s rho	65	r_s_ = 0.073	*p* = 0.562
The length of time a clinical educator has undertaken the mentoring role will result in higher capacity to engage scores	Spearman’s rho	64	r_s_ = 0.051	*p* = 0.690
Higher levels of banding will result in higher capacity to engage scores	Kruskal-Wallis test	66	H(3) = 1.55	*p* = 0.671
The level of academic qualification will affect capacity to engage scores	Kruskal-Wallis test	65	H(4) = 4.97	*p* = 0.290
Attainment of clinical educator training will impact on capacity to engage scores	Kruskal-Wallis test	66	H(4) = 1.34	*p* = 0.855
	Mann–Whitney U	66	U = 485.50	*p* = 0.796

## Results

### Response rate

The response rate was 42 % (*n* = 66) from an estimated 158 clinical educators. Of the 66 respondents to the survey 23 % (*n* = 15) were male and 77 % (*n* = 50) were female (one unknown). This ratio of approximately 1:3 (male: female) reflects the professional trend (Health and Care Professions Council 2012; personal email). Of the 66 respondents, 18 % (*n* = 12) were between 20 to 29, 24 % (*n* = 16) between 30 to 39, 32 % (*n* = 21) between 40 to 49 and 26 % (*n* = 17) between 50 to 59 years of age.

### Hypotheses testing

Statistical tests were conducted to test hypotheses relating to factors associated with podiatrists’ capacity to engage in clinical education. A number of significant results were obtained and are detailed in Table 1. Increased capacity to engage in clinical education was found to be associated with clinical educators volunteering for the role (Mann–Whitney U test, *p* = 0.003); full-time employment of the clinical educator (Mann–Whitney U test, *p* = 0.010); time being allocated to the clinical educator for preparation in advance of the student joining the placement (Mann–Whitney U test, *p* = 0.002); protected time for clinical education within the working day but outside clinical hours (Mann–Whitney U test, *p* = 0.002); mentors having a number of students, rather than a single student, to mentor (Mann–Whitney U Test, *p* = 0.037); responsibility to sign off students’ learning outcomes (Mann–Whitney U test, *p* = 0.006); having a relationship with the university outside the clinical educator role, such as having been a student there previously (Mann–Whitney U test, *p* ≤ 0.05); and proximity of the placement to the university (Kruskal-Wallis test, *p* ≤ 0.05).

A prior relationship with the University and the distance from the University both approached significance and to explore whether either had any potential explanatory value in predicting clinical educator capacity to engage with the role within the regression model the significance level was raised to *p* ≤ 0.1. Ten Mann–Whitney U tests were performed for each category resulting in ten paired independent samples. The significance level was relaxed and set at *p* ≤ 0.1 and the results were significant for four of the paired independent samples; 0 to 49 miles and 50 to 99 miles (*p* < 0.046); 0 to 49 miles and 100 to 149 miles (*p* < 0.046); 0 to 49 miles and 150 to 199 miles (*p* < 0.096); 0 to 49 miles and 200 to 249 miles (*p* < 0.063). Although, the results for distance from the university were not all significant at *p* ≤ 0.05 they were at *p* ≤ 0.1 and these four variables were included within the regression analysis in order to determine whether they had any explanatory value for predicting capacity to engage with clinical education within the regression model.

### Multiple linear regression

In total eleven variables were considered within the initial regression, subsequently producing a model comprised of four variables. Initially, the variables were considered together which led to the identification of one variable that was best able to predict the outcome based on levels of significance. The chosen variable was then retained within the model and a second predictor variable was subsequently identified. This process was repeated until all the variables had been either included or excluded from the regression model. This enabled the generation of a model to ascertain the extent to which the variables identified from the initial analysis were predictive of the variability of the dependent variable, ‘*clinical educator capacity to engage in the mentorship role’*. The results are presented in Table 3. The regression model summary produced was as follows: R_2_ 0.428 (*p* < 0.001).

**Table 3:** Multiple regression to identify predictors of podiatrists’ capacity to engage in clinical education

	B	SE.B	β
Step 1			
Constant	247.96	4.08	
Protected mentorship time	31.48	9.00	0.42*
Step 2			
Constant	242.28	4.21	
Protected mentorship time	32.80	8.36	0.44**
Clinical educator relationship with university	26.10	8.36	0.35**
Step 3			
Constant	227.96	6.13	
Protected mentorship time	27.01	8.01	0.36**
Clinical educator relationship with university	26.91	7.80	0.36**
Sign-off responsibilities	21.70	7.13	0.33**
Step 4			
Constant	221.47	6.40	
Protected mentorship time	25.52	7.67	0.34**
Clinical educator relationship with university	27.71	7.45	0.37**
Sign-off responsibilities	19.00	6.89	0.29**
Volunteer status of clinical educator	15.51	6.18	0.25**

Note: Adjusted R_2_ = 0.17 for Step 1 *p ≤ 0.001, adjusted R_2_ = 0.28 for step 2, adjusted R_2_ = 0.37 for step 3, adjusted R_2_ = 0.43 for step 4. **p < 0.001

The four independent variables identified (protected mentorship time, clinical educator relationship with university, sign-off responsibilities and volunteer status of clinical educator) represent 43 % of the predictive variability of the dependent variable - capacity to engage in clinical education.

## Discussion

This research aimed to identify the factors that impact upon clinical educators’ capacity to engage in the role in the context of podiatry. Findings revealed factors which increase the capacity of clinical educators in this role to include: being provided with protected time to engage in preparation and support of students; having a current or previous relationship with the university which goes beyond the clinical educator role; having assessment and sign-off responsibilities for students; and volunteering for the role. In addition to identifying individual factors which influence the capacity of clinical educators to engage in the role, the research produced a model capable of predicting individual clinical educators’ performance in relation to capacity to engage. The model accounts for 43 % of the predictive variability of capacity of clinical educators to engage with the role and, therefore, has utility in identifying opportunities for placement planning, organisation and support - resulting in more effective practice placement.

The findings of this study are supportive of Jokelainen et al. [13] who found protected time to be valued by clinical educators. The mentorship role is a major responsibility for the clinical educator both in terms of the student’s placement experience and their progression within the clinical environment. Ideally time should be embedded within the timetable for the clinical educator and student, outside the podiatrist’s clinical responsibilities, to engage with mentoring. This may include reflecting on the day’s or week’s events to contextualise experiences and reinforce theory, providing pastoral support and setting new goals and learning opportunities in partnership with the student.

This study has shown that where the clinical educator has a previous or existing relationship with the university, capacity for mentorship is increased. This result supports previous work where loyalty links have been established with a place of previous study or where endeavours which result in the attainment of an award are currently being undertaken [21]. This type of allegiance can be conceptualised as brand loyalty, with the University representing the brand. The students’ relationship with ‘using the brand’ appears to create a sense of loyalty which extend to actions beyond graduation [32].

Where clinical educators undertake the responsibility for signing-off learning outcomes there was found to be an increase in capacity for the role [6]. Assessment of competency is integral to the role and often necessitates liaison with other clinical educators regarding their assessment of student capabilities conferring considerable extra responsibility to the clinical educator who will decide on students’ ability to progress. The placement process may be more challenging for some students than for others, and ultimately be more rewarding for the clinical educator when a successful achievement of summative assessment is reached. Where clinical educators are not given this responsibility it may have a negative effect, with the clinical educator having spent time developing a student, but without recognition of this substantial investment.

Volunteering for the clinical educator role increased capacity scores. It would seem natural that individuals that choose to undertake a role are more likely to be well disposed towards it, as it is perhaps viewed as vocational rather than compulsory [32]. A requirement for an increase in placements allocations may result in staff having to take on these roles. This may be counter-productive as unwilling staff are unlikely to mentor students effectively, and may even impact on student attrition.

The insignificant results concerning the clinical educator’s education and experience are surprising, especially given evidence in other professions of the importance of qualifications on student learning (e.g. Nasr et al. [33]. This finding may reflect the homogeneity of variance associated with the sample which may not be reflective of samples in previous research. It is possible that less experienced staff are better able to understand the perspective of students than their more experienced colleagues, thus they off-set a lack of experience with an increased enthusiasm for the role.

The CECE scale provides a useful tool to examine the engagement of clinical educators in students’ learning. Further research using the scale with podiatrists both in the UK and internationally would provide important comparative data. The scale could also be adapted for use with other health professionals engaging in clinical education; this would be beneficial given the multi-professional context within which both practitioners and students frequently work. Building the body of work involving the CECE would yield larger samples thereby enabling more sophisticated statistical analyses with greater power. Given that the CECE scale is self-report, it would be important for future research to also measure aspects of the clinical environment independently (e.g. the ratio of educators to students; clinical caseload; student feedback).

Further research to explore other factors that impact upon capacity to engage in the role of clinical educator is required which surveys all podiatrists who undertake clinical education. Other possible factors which affect capacity could be included, such as the total number of students mentored each year by an individual clinical educator, perhaps from other health professions or universities and possibly on an ad hoc basis. Factor and Rasch analysis with a larger number of respondents may then be possible, to further develop and validate the CECE scale. Further testing of the model would also be beneficial. The CECE scale could be adapted and utilised with other healthcare professionals, increasing the sample size and inclusive of an international perspective. There is also scope to include other dimensions in the CECE scale, such as clinical educators’ perception of the responsibilities and ambit of the role. This work has the potential to provide guidance to the organisation and to inform the resourcing of healthcare students’ placements more generally.

### Limitations

While this research has contributed new knowledge in the area of podiatry training, the study suffers a number of limitations. First, the sample comprised of podiatrists from a single region of the UK, which limits the generalisability of the findings. Although the sample was drawn across both rural and urban placement contexts within a range of organisational environments of varying sizes, it is possible that regional variation may impact clinical education practice. Second, while the response rate to the survey was satisfactory, it is possible that non-respondents may have differed from respondents in relation to characteristics that were relevant to capacity to engage. The research findings, therefore, need to be interpreted with some caution. As with much survey research of this nature, the study assessed the perceptions of clinicians as to the barriers and facilitators to their engagement with the clinical educator role. Such perceptions are important as they describe the lived experience of clinicians and will affect their practice. Nevertheless, perceptions are not necessarily accurate reflections of the external environment.

## Conclusions

Establishing the factors that are significant in influencing capacity to undertake the role of clinical educator is crucial in the further support and development of placements in higher education. Capacity-building requires a sustainable approach with participation at an organisational, group and individual level, impacting upon management of placements at both a local and national level. This study has specifically focused on the individual, and on relationships between the university and practice setting. Commitment to investment of resources and opportunities is required, not only to increase individual capacity, but also to support quality and effectiveness of training opportunities. Enhancing clinical educator capacity for the role will promote the development of effective placements leading to the potential for increasing allocations and impacting positively upon attrition rates. At a practical level, this research informs podiatry placement recruitment to the role of clinical educator, which will promote engagement with the task. These findings are of relevance in relation to strategic planning, policy-making in the NHS and for the higher education institutions organising placements at a local level.

## Abbreviations

ART: action research team
CECE: clinical educator capacity to engage
NHS: National Health Service
UK: United Kingdom
